# Supplementary material for: Rapid evolution of genes with anti-cancer functions during the origins of large bodies and cancer resistance in elephants
Source: bioRxiv. 2024 Feb 29:2024.02.27.582135. Preprint. [Version 1] doi: 10.1101/2024.02.27.582135 (PMC10925141; doi:10.1101/2024.02.27.582135)
Supplement: Supplement 4 [file NIHPP2024.02.27.582135v1-supplement-4.pdf]

bioRxiv preprint doi: https://doi.org/10.1101/2024.02.27.582135; this version posted February 29, 2024. The copyright holder for this preprint (which was not certified by peer review) is the author/funder, who has granted bioRxiv a license to display the preprint in perpetuity. It is made available under aCC-BY 4.0 International license.

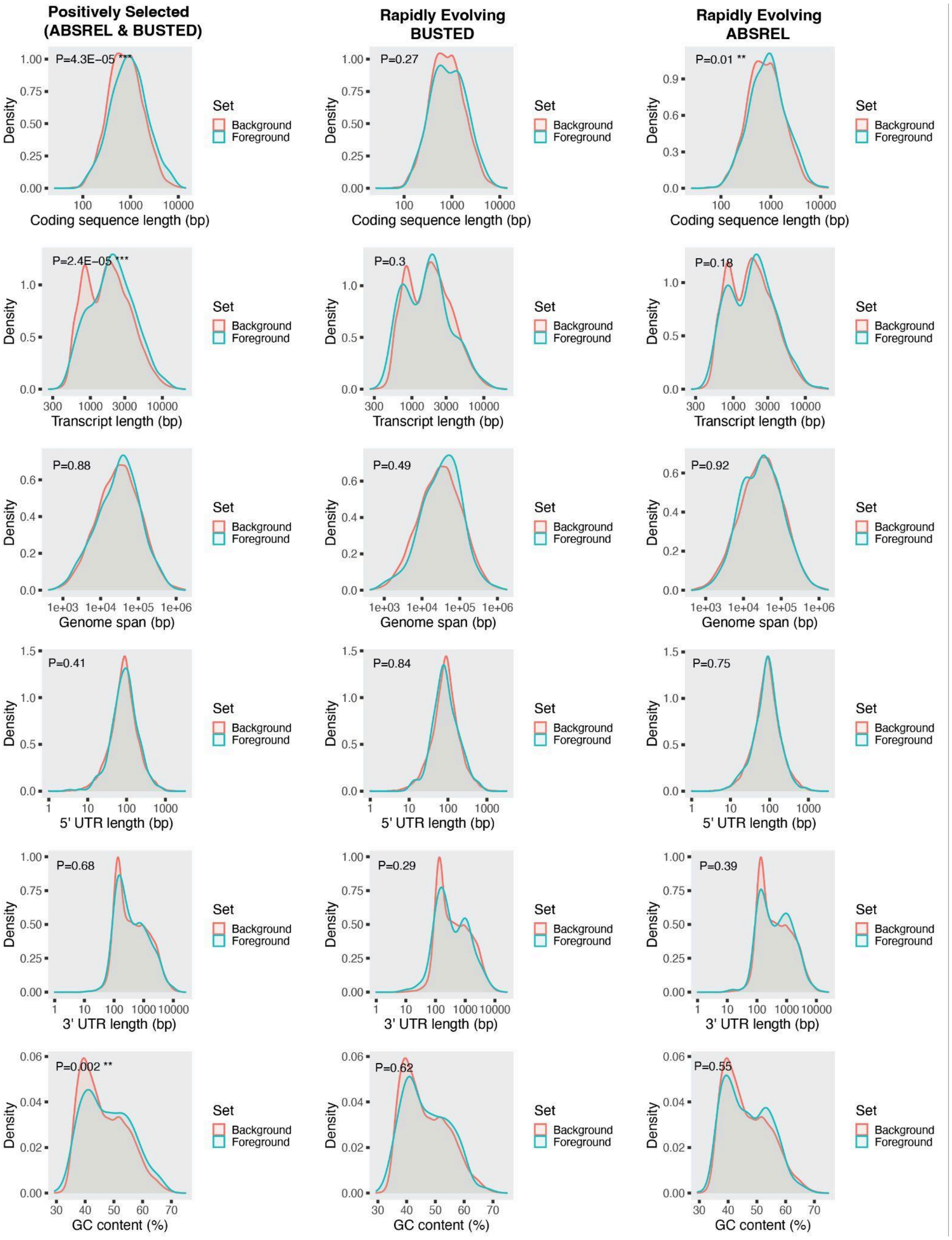

Figure 1 – figure supplement 1. Genomic features of positively selected and rapidly evolving genes (foregroundf) compared to all genes tested (Background). *P*-values are derived from a Chi-square test.
